# A New Digital Analysis Technique for the Mechanical Aperture and Contact Area of Rock Fractures

**DOI:** 10.3390/ma16041538

**Published:** 2023-02-12

**Authors:** Yong-Ki Lee, Chae-Soon Choi, Seungbeom Choi, Kyung-Woo Park

**Affiliations:** 1 Disposal Performance Demonstration Research Division, Korea Atomic Energy Research Institute (KAERI), 111 Daedeok-daero, 989 Beon-gil, Yuseong-gu, Daejeon 34057, Republic of Korea; 2 Disposal Safety Evaluation Research Division, Korea Atomic Energy Research Institute (KAERI), 111 Daedeok-daero, 989 Beon-gil, Yuseong-gu, Daejeon 34057, Republic of Korea

**Keywords:** rock fractures, mechanical aperture, contact area, digital analysis, pressure film

## Abstract

In this study, a new digital technique for the analysis of the mechanical aperture and contact area of rock fractures under various normal stresses is proposed. The technique requires point cloud data of the upper and lower fracture surfaces, pressure film image data of the fracture, and normal deformation data of the fracture as input data. Three steps of algorithms were constructed using these input data: (1) a primary matching algorithm that considers the shape of the fracture surfaces; (2) a secondary matching algorithm that uses pressure film images; and (3) a translation algorithm that considers the normal deformation of a fracture. The applicability of the proposed technique was investigated using natural fracture specimens sampled at an underground research facility in Korea. In this process, the technique was validated through a comparison with the empirical equation suggested in a previous study. The proposed technique has the advantage of being able to analyze changes in the mechanical aperture and contact area under various normal stresses without multiple experiments. In addition, the change in the contact area on the fracture surface according to the normal stress can be analyzed in detail.

## 1. Introduction

A growing interest in engineering projects that utilize underground spaces, such as deep mining, enhanced geothermal energy systems, geological disposal of CO_2_, and high-level radioactive waste disposal, has highlighted the importance of understanding the hydro-mechanical behavior of rock masses. This is because the groundwater and rock masses inevitably interact in underground spaces. As a result, the importance of rock fractures, which are mechanically weak planes and primary flow paths of fluids, has also emerged. Rock fractures comprise various parameters, including roughness, aperture, and contact area, which constitute the void geometry governing the hydro-mechanical behavior of the rock mass [1]. The mechanical aperture and contact area are the main parameters of the void geometry. The mechanical aperture is a parameter defined as the average height difference between the upper and lower fracture surfaces, and the contact area is a parameter that refers to the area where the upper and lower fracture surfaces come into contact with each other and transfer stress. The size of the fracture surfaces vary, and the contact area is thus generally defined as the ratio of the total surface area. Due to the importance of these two parameters for the hydro-mechanical behavior of rock masses, various studies have been conducted to measure them.

First, studies related to the measurement of the mechanical aperture are divided into surface topography, injection, and casting approaches. The surface topography approach was first applied by Gentier [2] to measure the mechanical aperture by digitizing the shape of the fracture with a profiler moving across the surface. Since then, precise surface measurements have been made possible thanks to the development of laser scanning devices, which remain the most commonly used method. However, a matching algorithm for the upper and lower fracture surfaces is required to derive the mechanical aperture, and the validity of this algorithm must be supported. The injection approach involves the application of epoxy resin (or metal) via injection onto the fracture surface, which is then hardened under normal stress. Next, the mechanical aperture is measured using this hardened resin. Since Gale [3] first applied this approach, it has been utilized in subsequent studies [4,5]. After cutting, the specimen containing the hardened resin as well as the thickness and cross section of the resin are analyzed using optical microscopy. As a result, this approach has the disadvantage of damaging the specimens [6]. In addition, the precision of the measurements is affected by the physical properties of the resin. Alternatively, the casting approach measures the mechanical aperture by creating transparent replicas of the fracture aperture space and then analyzing the images taken. This approach has been utilized in several studies [7,8,9]; however, it has disadvantages, such as damaging the specimen and a relatively low precision [6]. Approaches using X-ray CT have also been studied [10], which have the advantage of directly measuring the mechanical aperture of fractures. However, despite their considerable cost, these approaches have a lack of precision compared with the surface topography approach, which uses recently developed laser scanning devices.

Next, studies related to the contact area have mainly introduced methods of inserting a specific material into the fracture. Early studies measured the contact area by inserting a deformable plastic material between the fracture surfaces and applying a normal load [11]. Similarly, approaches have been introduced that insert a layer of low-viscosity casting epoxy [12] or a layer of white tempera with a black ink film [13]. However, these approaches have the disadvantage of overestimating the contact area because of the thickness of the insertion material. Recently, several studies have focused on measuring the contact area by performing compression tests, in which a pressure film is inserted instead of these materials. A pressure film has the advantage of enabling a detailed analysis as it can consider even the stress level acting on the contact area [14]. An approach that uses electrical current resistance has also been proposed [15], but this has a fatal disadvantage in that the measurement precision cannot be guaranteed, owing to the high current resistance of the rocks. In addition, techniques for analyzing the contact area of fractures using point cloud data have been proposed with the recent development of laser scanning devices [16,17]. These techniques are characterized by the fact that the matching algorithm of the upper and lower fracture surfaces affects the measurement result of the contact area, as in the case of the mechanical aperture.

In summary, using point cloud data obtained with a laser scanning device is the preferred approach for measuring the mechanical aperture and contact area. The use of a pressure film is preferred to measure the contact area. However, as the approaches in previous studies have been limited to measuring the mechanical aperture and contact area under a specific normal stress, only results under the normal stress applied in the measurement process can be analyzed. In other words, multiple measurements are required to analyze the changes in the mechanical aperture and contact area under various normal stresses.

In this context, this study proposes a new digital technique that can derive the mechanical aperture and contact area of fractures under various normal stresses by using point cloud data with both the pressure film data and normal deformation data of the fracture. The proposed technique consists of three steps: (i) a primary matching algorithm based on the shape of the fracture surfaces; (ii) a secondary matching algorithm using pressure film images; and (iii) a translation algorithm that considers the normal deformation of fractures. The applicability of the proposed technique was investigated using natural fracture specimens sampled at an underground research facility in Korea. In this process, the technique was validated through a comparison with the empirical equation suggested in a previous study.

## 2. Methodology of the Digital Analysis Technique

To construct a digital technique with which to analyze the mechanical aperture and the contact area of the rock fractures under various normal stresses, we attempted to utilize the advantages of the surface topography approach using a laser scanning device and the method using a pressure film. In addition, an algorithm using the normal deformation of rock fractures was applied to analyze the mechanical aperture and contact area according to the change in normal stress without additional experiments. The proposed technique derives the results in three steps. This chapter presents the method of preparing the input data for applying the technique and the algorithms used for each step.

### 2.1. Preparation of Input Data

The digital analysis technique requires three types of input data: (i) point cloud data for fracture surfaces; (ii) image data from a pressure film compression test; and (iii) normal deformation data from compression tests on intact and fractured rock specimens. The input data can be prepared using laser scanning and compression tests.

**Figure 1:**
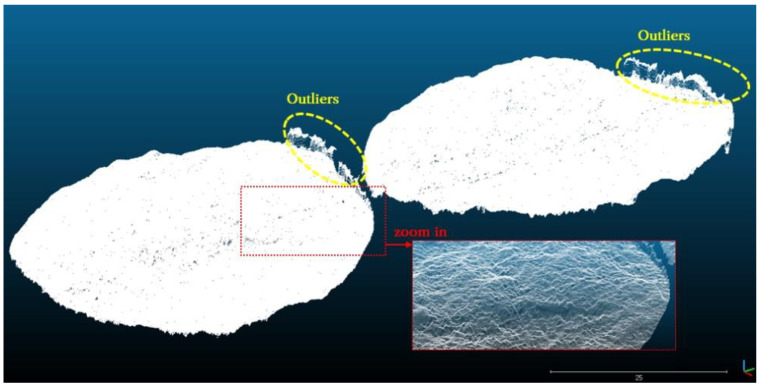
Example of point cloud data of fracture surfaces acquired using a laser scanning device.

First, point cloud data for fracture surfaces can be obtained using a commonly used laser scanning device. The higher the precision of the laser scanning device, the more advantageous it is. In particular, higher precision is required for the z-axis, which is the elevation direction. For the x- and y-axes, it is recommended to ensure measurement intervals of less than 0.5 mm. Three-dimensional point cloud data can be obtained by measuring the upper and lower fracture surfaces using a laser scanning device. Figure 1 shows an example of the point cloud data measured on the upper and lower surfaces. Outliers were observed at the fracture boundaries owing to the characteristics of laser scanning; therefore, a removal process was required during analysis.

**Figure 2:**
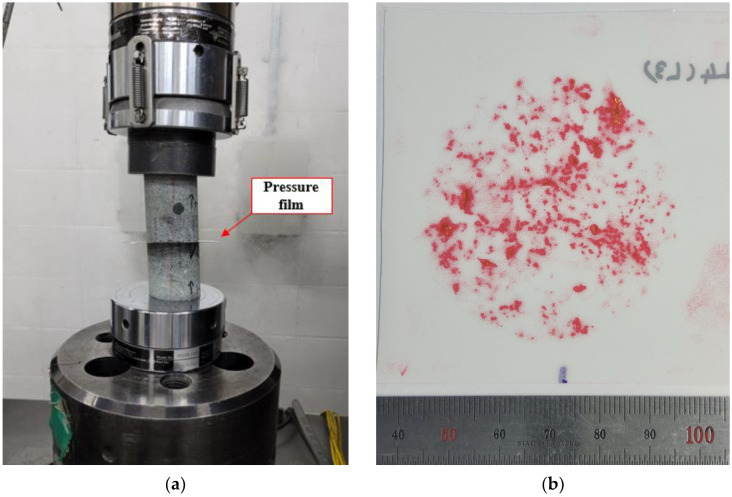
Example of pressure film image data acquisition: (**a**) pressure film compression test; (**b**) pressure film image data.

Second, the pressure film image data can be obtained by performing a compression test on a rock fracture in which a pressure film is inserted. The pressure film loaded to a specific normal stress is photographed using a digital camera to obtain the image data. After the pressure film is inserted between the fractures, care must be taken to ensure that the upper and lower surfaces are joined in conformance. In general, there is no problem unless the surface is significantly distorted because the fracture is well joined as the normal load begins to be applied. The applied normal stress should not be too high so that sufficient normal deformation of the fracture occurs. Considering the properties of general rock fractures, normal stress in the range of 3–5 MPa is appropriate. In addition, the pressure film should be capable of covering the low normal stress range. Figure 2 shows an example of a compression test using a pressure film and the image data obtained from it.

**Figure 3:**
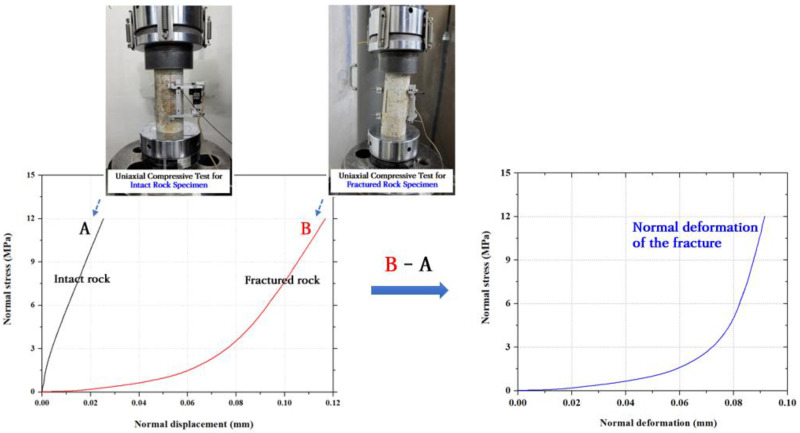
Schematic of data analysis to derive the normal deformation data of the rock fracture.

Third, normal deformation data for rock fractures can be obtained from compression tests on intact and fractured rock specimens [18]. After measuring the normal displacement of each specimen under various normal stress conditions, the normal deformation data of the fracture is derived by calculating the difference between the displacements of the fractured rock and intact rock under the same normal stress. Figure 3 shows a schematic representation of the data analysis to derive the normal deformation data for the rock fracture.

### 2.2. First Step: Primary Matching According to the Shape of Fracture Surfaces

Because the point cloud data is obtained by measuring the separated upper and lower surfaces of the rock fracture, it is necessary to match them to one fracture. An algorithm that matches the upper and lower surfaces considering the surface shape was applied as the first step. In this step, the iterative closest point (ICP) algorithm [19], widely used for matching different point cloud data, was mainly used. The ICP algorithm estimates the correspondences between two point cloud data and then aligns them until the distance error is below the threshold. In estimating correspondences, pairs of points with the closest distance from each point cloud data are used. Assuming that there are different point cloud data *P*_1_ and *P*_2_, the flow of the ICP algorithm is briefly summarized as follows:

- The correspondences of *P*_1_ to *P*_2_ are constructed by searching for the points of *P*_2_ closest to each point of *P*_1_.
- A rotation matrix (*R*) and translation vector (*t*) are derived based on the correspondences.
- *P*_1_ is updated to $\bar{P_{1}}\left(=R\times P_{1}+t\right)$, then aligned with *P*_2_.
- The above process is repeated until the point-to-point distance error between the $\bar{P_{1}}$ and *P*_2_ is smaller than the threshold.

**Figure 4:**
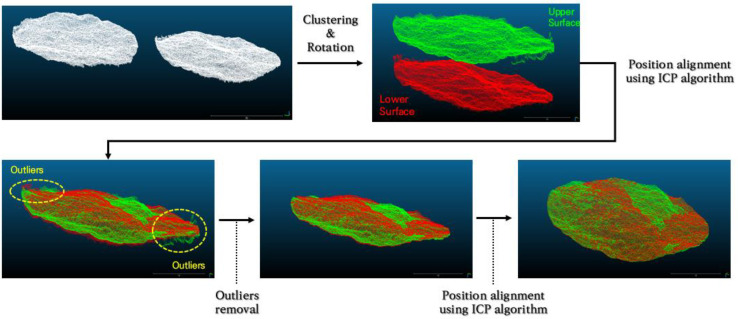
Schematic of an algorithm for primary matching according to the shape of the fracture surfaces.

The first step starts with clustering the measured point cloud data based on distance and dividing it into two sets of point cloud data composed of points corresponding to the upper and lower fracture surfaces. Then, the points corresponding to the upper surface are rotated to match the approximate position, and the ICP algorithm is applied. Next, the outliers are removed by setting a region of interest so that outliers are not included. The ICP algorithm is reapplied to the two point cloud data, from which outliers have been removed. This is because, in the first matching using the ICP algorithm, the matching accuracy is lowered owing to the influence of outliers. A schematic of the primary matching algorithm is shown in Figure 4.

### 2.3. Second Step: Secondary Matching Using Pressure Film Image

**Figure 5:**
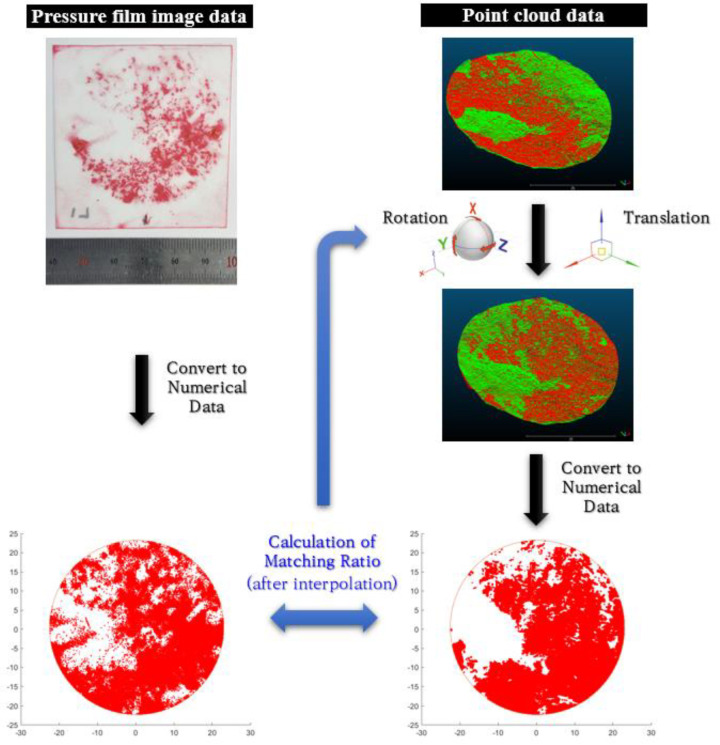
Schematic of an algorithm for secondary matching using the pressure film image.

The matched point cloud data in the first step is not under normal stress conditions because only the shape of the fracture surfaces is considered. This study used pressure film images to match the upper and lower fracture surfaces under specific normal stress conditions. As described in Section 2.1, because the pressure film image data is obtained by applying a load up to a specific normal stress, the contact area at that normal stress appears in the image. Therefore, if the point cloud data are adjusted such that the contact area coincides with the pressure film image data, the point cloud data can represent the state of fracture under the normal stress condition applied to the pressure film. In this study, a secondary matching algorithm was constructed to adjust the point cloud data to the pressure film image. Figure 5 shows a schematic of the secondary matching algorithm, which is summarized as follows:

- The pressure film image obtained by applying specific normal stress is converted into numerical data via image processing. Because only the identification of the contact area is of interest, the presence or absence of contact is determined without considering the intensity of the image.
- The contact area of the point cloud data is converted into numerical data. If the coordinates of the upper surface are located lower than those of the lower surface, it is determined as a contact state, and the coordinates of the upper surface are changed to be identical to those of the lower surface.
- Interpolation is performed such that the numerical data of the pressure film and point cloud data are located on the exact grid coordinates.
- The matching ratio is calculated by comparing the two numerical datasets. The matching ratio refers to the percentage of grids that match contact/non-contact to the total number of grids.
- The above process is repeated while the point cloud data of the upper surface is rotated and translated about the x-, y-, and z-axes, and the matching ratio is calculated under all conditions.
- Subsequently, the condition where the matching ratio is minimized is assigned as the state under the normal stress applied in the pressure film compression test.

Because the point cloud data is already primarily matched, it is not necessary to include a wide range of rotations and translations in the secondary matching algorithm. For each axis, it is sufficient to apply ±3° rotation and ±3 mm translation. As the z-axis, which is the elevation direction, is affected by the normal deformation of the fracture, it is recommended to apply a range of about ±5 mm along the z-axis. The smaller the rotation and translation interval within the range, the higher the reliability of the result. Therefore, considering the computational efficiency, it is better to set the interval as small as possible. In general, reasonable results were obtained at a rotation interval of about 0.1° and a translation interval of about 0.01 mm.

### 2.4. Third Step: Translation Considering the Normal Deformation of Fracture

The point cloud data matched to the pressure film image shows the results under the normal stress condition applied in the pressure film compression test. The normal deformation of the fracture caused by normal stress should be considered to analyze the change in the mechanical aperture and contact area under various normal stress conditions. In this study, a translation algorithm using the normal deformation data of rock fractures was applied to calculate the mechanical aperture and contact area under various normal stress conditions. The algorithm is as follows:

- The normal stress applied in the pressure film compression test is assigned as the reference normal stress. Then, the difference in the normal displacement of the fracture caused by the change from the reference normal stress to the target normal stress is calculated using the normal deformation data.
- The point cloud data on the upper surface of the fracture are translated in the z-axis direction using the difference in the normal displacement. If the coordinates of the upper surface are located below the coordinates of the lower surface during translation (the elevation difference is negative), it is determined to be a contact, and the coordinates of the upper surface are set to the same value as the coordinates of the lower surface. When the elevation difference changes from negative to positive due to translation, the upper surface coordinates are restored by considering the original shape.
- After the translation, the mechanical aperture and contact area of the fracture are calculated. The average value of the elevation differences at each grid point is derived as the mechanical aperture, and the ratio of the number of contacted grids to the total number of grids is derived as the contact area ratio.

**Figure 6:**
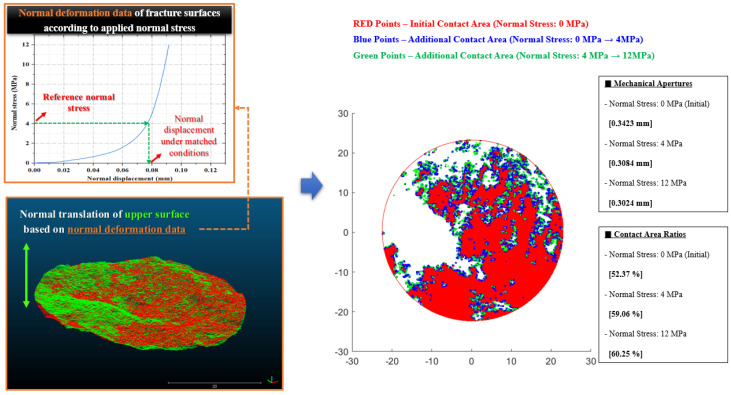
Example of the application result of the translation algorithm considering a normal deformation of fracture.

Figure 6 shows an example of the application of the translation algorithm. The left side of the figure shows a schematic of the algorithm, and the right side shows the results derived from this. The example results in the figure correspond to a normal stress of 0 MPa as the initial condition, 4 MPa as the reference condition, and 12 MPa as the condition of approximately 500 m depth. The blue and green points show the changes in the contact area according to each condition.

## 3. Applicability of the Proposed Technique

The applicability of the proposed technique was examined using natural fracture specimens sampled from the KAERI underground research tunnel (KURT), an underground research facility in Korea. KURT was built to develop disposal technologies for high-level radioactive waste and to verify the performance of the disposal system. Natural fracture specimens for applicability examination were sampled from a deep borehole with various rock types. In this section, the results of the applicability examination with the experimental procedure are presented, including information on the sampled specimens. In addition, the analysis of the change in the mechanical aperture and contact area according to the normal stress of the sampled specimens using the proposed technique is also presented.

### 3.1. Experimental Procedure

**Figure 7:**
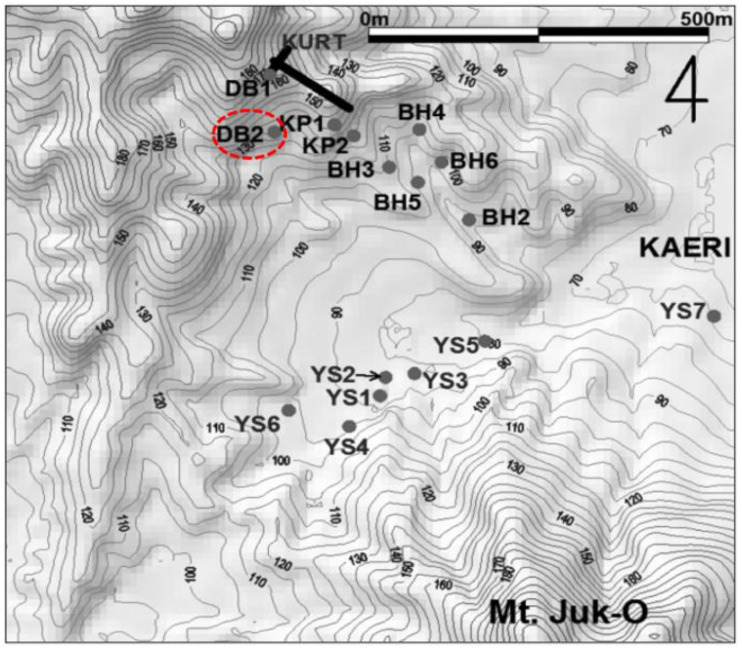
Location map for the DB-2 borehole.

A total of 12 natural fracture specimens were sampled from deep boreholes (DB-2) in KURT to examine the applicability of the proposed technique. The DB-2 borehole is located in Daejeon, Korea, as shown in Figure 7.

To analyze the applicability at different rock types and depths, granite, altered rock, and fine-grained andesite dyke specimens were sampled at low and high depths. Because KURT is related to the disposal of high-level radioactive waste, it was divided into low and high depths based on a disposal depth of 500 m. The types of sampled specimens and their notations used in this study were as follows:

- Granite at a depth of 250 m: GL.
- Granite at a depth of 840 m: GH.
- Altered rock at a depth of 320 m: AR.
- Fine-grained andesite dyke at a depth of 750 m: FAD.

**Figure 8:**
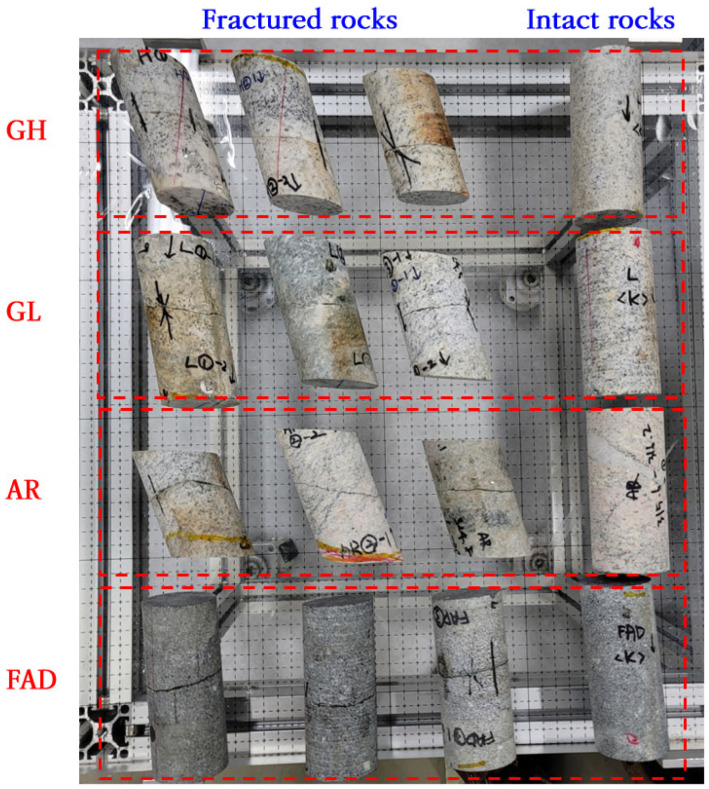
Intact and fractured rock specimens sampled to investigate the applicability of the proposed technique.

As shown in Figure 8, three specimens were sampled for each type, and intact rock specimens were prepared to obtain normal deformation data of the rock fracture. In addition, the physical and mechanical properties were analyzed using additional intact rock specimens, and the results are listed in Table 1.

**Figure 9:**
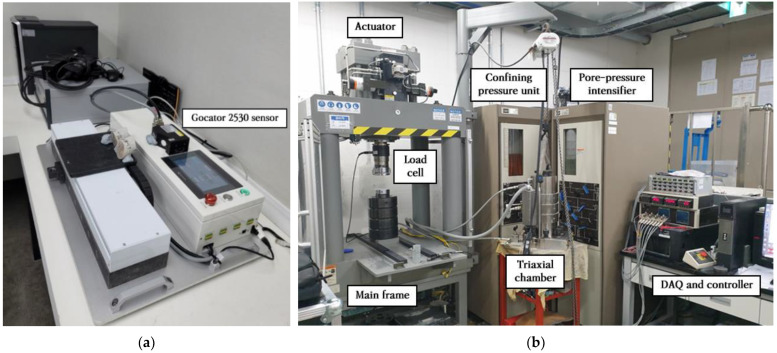
Test equipment used to investigate the applicability of the proposed technique: (**a**) 3-D laser scanning device; (**b**) MTS 816 system.

The laser scanning device for acquiring point cloud data of the fracture surfaces used the equipment shown in Figure 9a, manufactured by SPOS Corporation. This equipment includes the LMI technologies’ Gocator 2530 sensor, enabling elevation measurement with a resolution of 0.001 mm at intervals of 0.03–0.05 mm. The MTS 816 system (as shown in Figure 9b), which is widely used for rock testing, was used as the compression test equipment to acquire the normal deformation data of the fracture as well as the physical and mechanical properties of the specimens.

The pressure film used for acquiring the pressure film image data was Fujifilm Corporation’s Prescale super low. This film is a two-sheet type, and a detailed classification is possible up to normal stress of 2.5 MPa. This type of film showed normal stresses exceeding 2.5 MPa with the same intensity. However, because the proposed technique does not consider intensity, this film that covered low normal stresses in detail was suitable. The pressure film compression test applied a load up to a normal stress of 4 MPa using this film.

### 3.2. Validation of the Proposed Technique

The applicability of the proposed technique was validated using the prepared specimens. The matching algorithm (first and second steps) was examined using the matching ratio with the pressure film image data, which were related to the validation of the contact area analysis. The result of the mechanical aperture derived by applying the entire algorithm was compared with the empirical equation of a previous study for validation.

**Figure 10:**
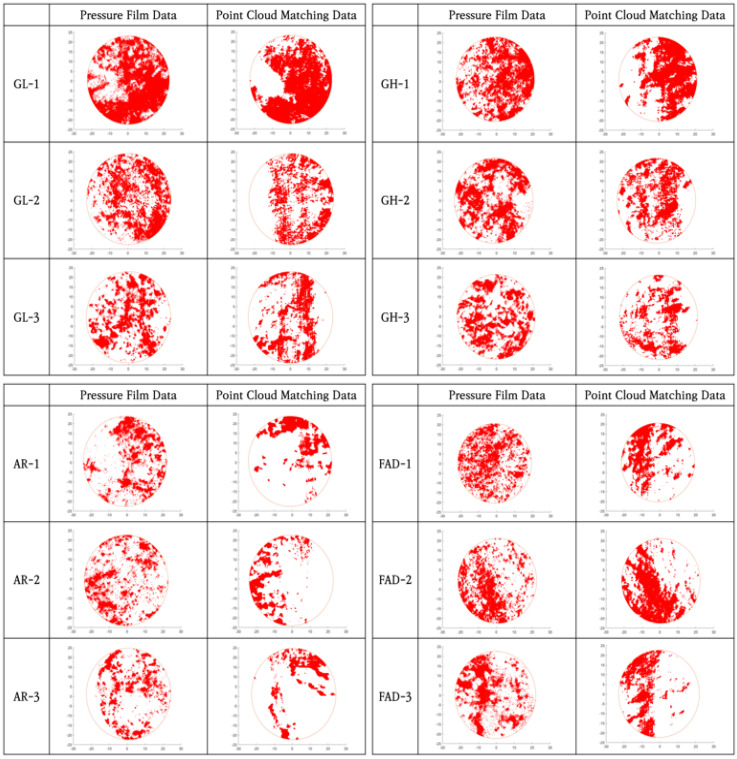
Comparison result of numerical data through the application of the matching algorithm.

Figure 10 shows the results obtained by applying the matching algorithm to the 12 natural fracture specimens. The numerical data of the matching result (point cloud matching data) and numerical data of the pressure film image (pressure film data) are shown together in the figure.

The analysis results for the matching ratio and the contact area are listed in Table 2. The contact area was analyzed using the contact area ratio, which is the ratio of the contact area to the area of the entire fracture surface. This result corresponded to the reference normal stress condition of 4 MPa applied in the pressure film compression test.

The average matching ratio was approximately 70%, and the lowest value was approximately 62%. However, the distribution of the contact area was similar, as shown in Figure 10, and the contact area ratio exhibited an average difference of 3.89%, as shown in Table 2. These results indicate that there were no significant problems with the application of the matching algorithm.

The mechanical aperture derived using the proposed technique was compared with an empirical equation from a previous study. Most studies have aimed to measure the mechanical aperture under a specific normal stress; therefore, they have mainly dealt with the initial mechanical aperture (*e*_0_) under zero normal stress. Therefore, the empirical formula for the initial mechanical aperture proposed by Bandis et al. [18] was used for comparative analysis. This empirical equation is defined in Equation (1), and has been widely used because it is composed of general fracture parameters:

(1) $$e_{0}=\frac{JRC}{5}\left(0.2\frac{\sigma_{c}}{JCS}-0.1\right)$$

where *JRC*, *σ_c_*, and *JCS* are the joint roughness coefficient, uniaxial compressive strength, and joint wall compressive strength, respectively.

*JRC* was calculated through its relationship with *Z_2_* (the root mean square of the first derivative of the profile), as in Equation (2) suggested by Tse and Cruden [20]. Profiles were extracted at intervals of 0.1 mm, and the average *Z*_2_ value was used. For the calculation interval of *Z*_2_, 0.5 mm belonging to the appropriate interval range suggested in the previous study [21] was applied. Table 3 lists the calculation results of the *JRC* of the upper and lower surfaces of the specimens, and the average value was used as the *JRC* of the specimen.

(2) $$JRC=32.2+32.47log\left(Z_{2}\right)\;$$

where

(3) $$Z_{2}=\sqrt{\frac{1}{L}\int_{0}^{L}{\left(\frac{dy}{dx}\right)}^{2}dx}=\sqrt{\frac{1}{L}\overset{N-1}{\underset{i=1}{\sum }}\frac{{\left(y_{i+1}-y_{i}\right)}^{2}}{x_{i+1}-x_{i}}}\;$$

The *JCS* of unweathered rocks is known to be equal to the uniaxial compressive strength [22], and so *σ_c_*/*JCS* was substituted with one for the GL, GH, and FAD specimens of fresh surfaces. The AR specimens are altered rocks with surface weathering. Therefore, *JCS* was measured using a Schmidt hammer, and, as a result, a value of 1.54 was substituted into *σ_c_*/*JCS*.

The initial mechanical apertures derived by applying the empirical equation and digital analysis technique proposed in this study are presented in Table 4. In the digital analysis technique, the results under a normal stress condition of 0 MPa were derived by considering the normal deformation from the reference normal stress condition of 4 MPa. The results show an average difference of 0.0319 mm and an error rate of 10.68%.

**Figure 11:**
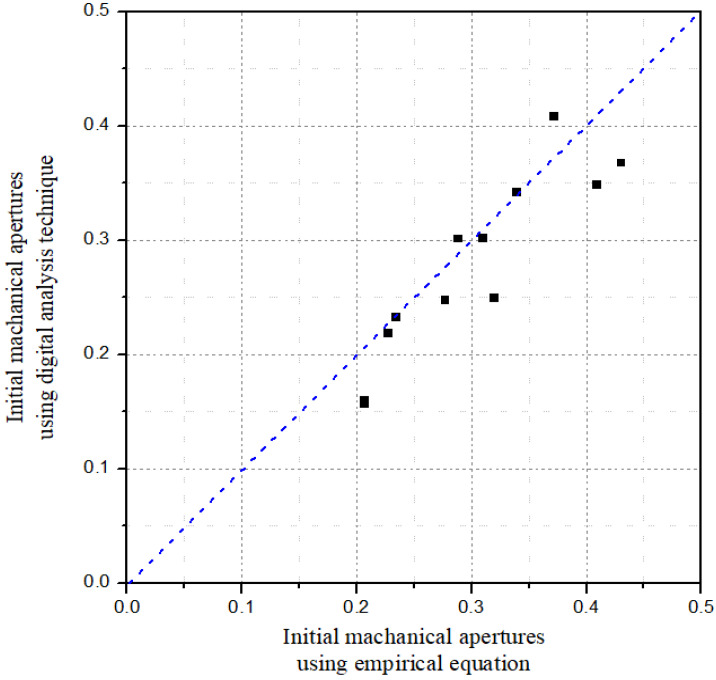
Result of the comparison between initial mechanical apertures derived using the empirical equation and digital analysis technique.

Figure 11 compares the two results and shows that the digital analysis technique can reasonably derive the initial mechanical aperture. However, the digital analysis method showed a slight underestimation of the initial mechanical aperture compared with the empirical equation. This is because the normal deformation of the fracture was slightly affected by the insertion of the pressure film. Better results could be obtained if the effect of the pressure film on the normal deformation of the fracture was considered.

### 3.3. Application to Natural Fractures at the KURT Site

The proposed technique was applied to the prepared specimens to examine the applicability in the analysis of the mechanical aperture and contact area according to the normal stress variation. In general rock fractures, the change in the mechanical aperture and contact area becomes small because the normal deformation rapidly decreases above specific normal stress. This tendency has also been observed in previous studies [23,24]. The natural fracture specimens at the KURT site sampled in this study showed a rapid decrease in normal deformation above 4 MPa.

**Figure 12:**
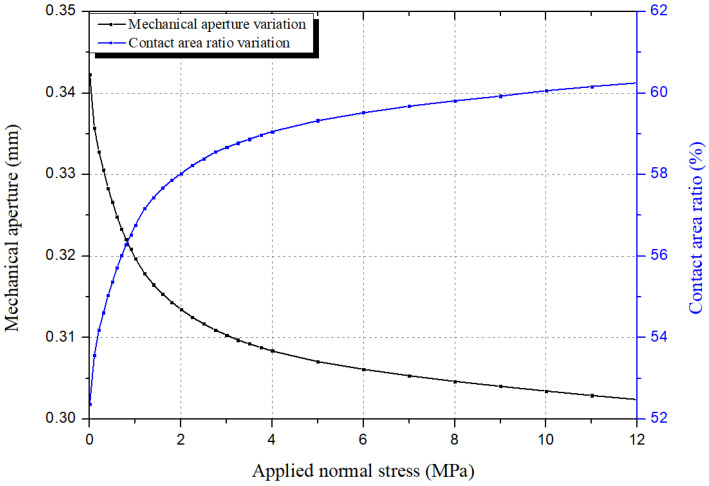
Variation of the mechanical aperture and contact area ratio under various normal stress conditions for the GL1 specimen.

Figure 12 shows the results of analyzing the change in the mechanical aperture and contact area under various normal stresses for the GL1 specimen, which showed similar results to other specimens. Table 5 summarizes the mechanical aperture and contact area ratio when the normal stresses of 0 MPa, 4 Mpa, and 12 Mpa were applied. The mechanical aperture and contact area ratio of all the specimens did not show significant differences when 4 Mpa and 12 Mpa were applied. This result indicates that the normal deformation change was not substantial when normal stress conditions of over 4 Mpa were applied.

**Figure 13:**
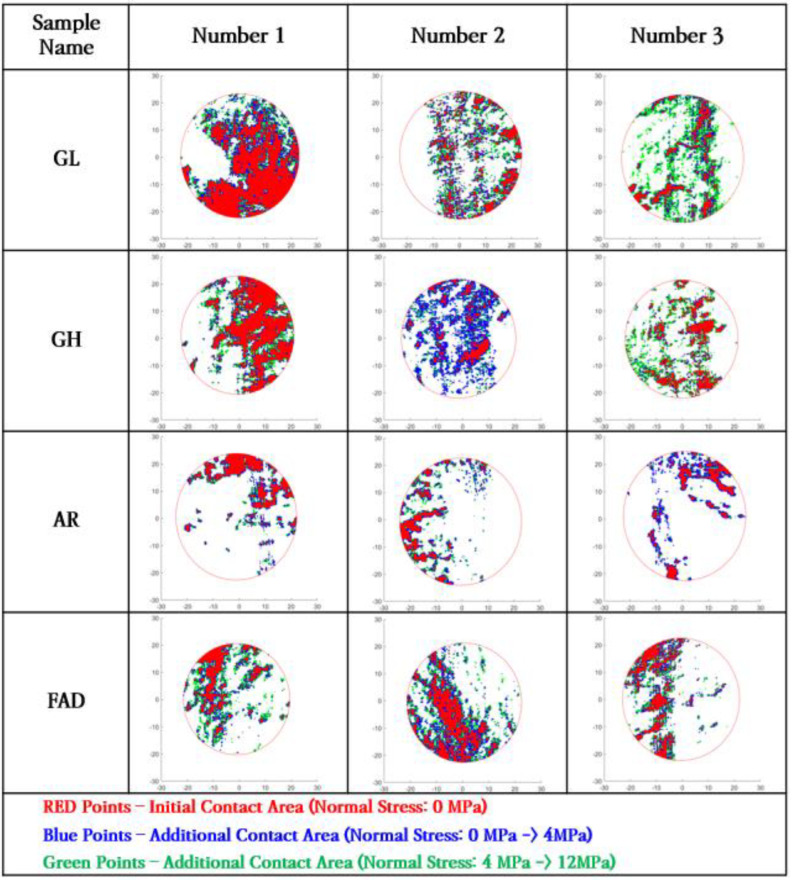
Changes in the contact area with increasing normal stress for natural fracture specimens.

Figure 13 shows the change in the contact area under various normal stresses in more detail. This figure shows the changes in the contact area as the normal stress condition increases to 0 MPa, 4 MPa, and 12 MPa (red, blue, and green points, respectively). As the normal stress increases, the area near the initial contact area gradually changed to the contact state.

These results indicate that the mechanical aperture and contact area, under various normal stress conditions in rock fractures, can be derived without additional experiments using the proposed digital analysis technique. In addition, the proposed technique has the advantage of being able to analyze the change in the contact area on the fracture surface in detail, as shown in Figure 13.

## 4. Conclusions

This study proposes a new digital analysis technique for the analysis of the mechanical aperture and contact area of rock fractures under various normal stress conditions. The proposed technique includes matching algorithms for point cloud data using pressure film image data and a translation algorithm using normal deformation data of the fracture. The applicability of the proposed technique was examined using natural fracture specimens from the KURT site, an underground research facility in Korea.

The main findings of this study are summarized as follows:

(1) A new algorithm was proposed to match the point cloud data of the upper and lower surfaces using the pressure film image data of rock fractures. This consisted of a primary matching algorithm using the ICP algorithm and a secondary matching algorithm using pressure film image data. The proposed matching algorithm was validated using the results of the contact area and the matching ratio of natural fracture specimens.
(2) A new algorithm was proposed to analyze the mechanical aperture and contact area according to the change in normal stress using the normal deformation data of rock fractures. The proposed algorithm was validated by deriving the initial mechanical aperture using matched point cloud data under reference normal stress and then comparing the results with the empirical equation of a previous study.
(3) A digital analysis technique was proposed by synthesizing the above algorithms. The types and acquisition methods of the input data required to apply the proposed technique were presented. In addition, the entire algorithm was organized sequentially.
(4) To examine its applicability, the mechanical aperture and contact area under various normal stresses were analyzed by applying digital analysis techniques to natural fracture specimens at the KURT site, an underground research facility in Korea. As the normal stress increased, the change in the mechanical aperture and contact area was found to decrease rapidly, and the area adjacent to the initial contact area gradually switched to the contact state.

This study is significant because it proposed a new technique for analyzing the mechanical aperture and contact area, which are considered important parameters in the hydro-mechanical behavior of rock mass. As in the cubic law [25], the aperture is a parameter that is generally required in the hydro-mechanical coupled analysis. The proposed technique has the main advantage of being able to analyze changes in the mechanical aperture and contact area under various normal stress conditions without additional experiments. In addition, the change in the contact area on the fracture surface according to the normal stress can be analyzed in detail.

However, when applying the proposed technique, noise due to the sensitivity of the pressure film affects the contact area and matching ratio results. In addition, the slight deformation of the pressure film affected the derivation of the mechanical aperture based on normal deformation. Therefore, the proposed technique should be supplemented in the future by selecting an appropriate pressure film or applying a correction according to the type of pressure film.

The technique and application results of this study are expected to be utilized in various underground space-related projects that require analyzing rock mass properties from borehole cores.

## Figures and Tables

**Table 1:** Physical and mechanical properties of the specimens used in this study.

	GL	GH	AR	FAD
Dry unit weight (kg/m^3^)	2637.68	2604.12	2602.46	2743.17
Porosity (%)	0.91	0.51	0.70	0.51
P-wave velocity (m/s)	3276.19	3783.51	4029.59	3593.14
S-wave velocity (m/s)	2484.88	2566.43	2723.80	2783.54
Uniaxial compressive strength (MPa)	184.73	161.46	129.42	135.61
Young’s modulus (GPa)	57.30	71.40	47.00	50.40
Poisson’s ratio	0.21	0.31	0.05	0.36

**Table 2:** Analysis result of matching ratio and contact area for natural fracture specimens.

Sample Name	Contact Area Ratio in Point Cloud Matching Data (%)	Contact Area Ratio in Pressure Film Data (%)	Difference in Contact Area Ratio (%)	Matching Ratio (%)
GL-1	59.06	48.08	10.98	66.16
GL-2	25.19	29.58	4.39	64.66
GL-3	24.63	22.75	1.88	66.44
GH-1	45.55	38.83	6.72	62.23
GH-2	28.51	32.25	3.74	61.74
GH-3	22.13	26.54	4.41	63.38
AR-1	17.03	16.33	0.70	77.29
AR-2	14.10	14.84	0.74	81.45
AR-3	12.51	13.30	0.79	78.14
FAD-1	26.23	26.35	0.12	63.62
FAD-2	35.74	24.06	11.68	70.75
FAD-3	20.07	19.50	0.57	75.94
Average			3.89%	69.32%

**Table 3:** Joint roughness coefficient values of natural fracture specimens.

Sample Name	Upper Surface	Lower Surface	Average
GL-1	16.29	17.57	16.93
GL-2	13.47	14.16	13.82
GL-3	11.48	11.88	11.68
GH-1	10.30	10.28	10.29
GH-2	15.21	15.73	15.47
GH-3	11.30	9.33	10.32
AR-1	8.82	9.04	8.93
AR-2	10.13	9.54	9.84
AR-3	10.28	10.40	10.34
FAD-1	15.19	13.59	14.39
FAD-2	15.21	16.70	15.96
FAD-3	11.73	10.94	11.34

**Table 4:** Initial mechanical apertures derived using the empirical equation and digital analysis technique.

Sample Name	Derived Initial Mechanical Aperture (mm)		Difference in Initial Mechanical Aperture (mm)	Error Rate Compared to Empirical Equation (%)
	Empirical Equation	Digital Analysis Technique		
GL-1	0.3386	0.3423	0.0037	1.09
GL-2	0.2763	0.2481	0.0282	10.21
GL-3	0.2336	0.2331	0.0005	0.21
GH-1	0.2058	0.1607	0.0451	21.91
GH-2	0.3094	0.3024	0.0070	2.26
GH-3	0.2063	0.1579	0.0484	23.46
AR-1	0.3709	0.4089	0.0380	10.24
AR-2	0.4085	0.3492	0.0593	14.52
AR-3	0.4295	0.3682	0.0613	14.27
FAD-1	0.2878	0.3019	0.0141	4.90
FAD-2	0.3191	0.2501	0.0690	21.62
FAD-3	0.2267	0.2189	0.0078	3.44
Average			0.0319 mm	10.68%

**Table 5:** Mechanical apertures and contact area ratios under various normal stresses for natural fracture specimens.

Sample Name	Mechanical Aperture (mm)			Contact Area Ratio (%)		
	σ*_n_* = 0 MPa	σ*_n_* = 4 MPa	σ*_n_* = 12 MPa	σ*_n_* = 0 MPa	σ*_n_* = 4 MPa	σ*_n_* = 12 MPa
GL1	0.3423	0.3084	0.3024	52.37	59.06	60.25
GL2	0.2481	0.228	0.2209	21.29	25.19	26.69
GL3	0.2331	0.1835	0.1655	17.15	24.63	27.98
GH1	0.1607	0.145	0.1392	41.8	45.55	46.97
GH2	0.3024	0.2291	0.2251	17.39	28.51	29.28
GH3	0.1579	0.1491	0.1425	20.02	22.13	23.85
AR1	0.4089	0.3858	0.3815	15.04	17.03	17.43
AR2	0.3492	0.32	0.315	11.18	14.1	14.68
AR3	0.3682	0.3357	0.334	9.95	12.51	12.68
FAD1	0.3019	0.2511	0.2392	20.23	26.23	27.88
FAD2	0.2501	0.1984	0.1894	28.67	35.74	37.11
FAD3	0.2189	0.1992	0.1929	17.59	20.07	20.89

## Data Availability

Not applicable.
